# Validation of ion mobility spectrometry ‐ mass spectrometry as a screening tool to identify type II kinase inhibitors of FGFR1 kinase

**DOI:** 10.1002/rcm.9130

**Published:** 2021-06-29

**Authors:** Helen S. Beeston, Tobias Klein, Richard A. Norman, Julie A. Tucker, Malcolm Anderson, Alison E. Ashcroft, Geoffrey A. Holdgate

**Affiliations:** ^1^ Astbury Centre for Structural Molecular Biology & Faculty of Biological Sciences University of Leeds Leeds LS2 9JT UK; ^2^ Discovery Sciences, BioPharmaceuticals R&D, AstraZeneca, Alderley Park Macclesfield SK10 4TG UK

## Abstract

**Rationale:**

The protein kinase FGFR1 regulates cellular processes in human development. As over‐activity of FGFR1 is implicated with cancer, effective inhibitors are in demand. Type I inhibitors, which bind to the active form of FGFR1, are less effective than type II inhibitors, which bind to the inactive form. Screening to distinguish between type I and type II inhibitors is required.

**Methods:**

X‐ray crystallography was used to indicate whether a range of potential inhibitors bind to the active or inactive FGFR1 kinase conformation. The binding affinity of each ligand to FGFR1 was measured using biochemical methods. Electrospray ionisation ‐ ion mobility spectrometry ‐ mass spectrometry (ESI‐IMS‐MS) in conjunction with collision‐induced protein unfolding generated a conformational profile of each FGFR1–ligand complex. The results indicate that the protein's conformational profile depends on whether the inhibitor is type I or type II.

**Results:**

X‐ray crystallography confirmed which of the kinase inhibitors bind to the active or inactive form of FGFR1 kinase. Collision‐induced unfolding combined with ESI‐IMS‐MS showed distinct differences in the FGFR1 folding landscape for type I and type II inhibitors. Biochemical studies indicated a similar range of FGFR1 affinities for both types of inhibitors, thus providing confidence that the conformational variations detected using ESI‐IMS‐MS can be interpretated unequivocally and that this is an effective screening method.

**Conclusions:**

A robust ESI‐IMS‐MS method has been implemented to distinguish between the binding mode of type I and type II inhibitors by monitoring the conformational unfolding profile of FGFR1. This rapid method requires low sample concentrations and could be used as a high‐throughput screening technique for the characterisation of novel kinase inhibitors.

## 1 INTRODUCTION

There are over 500 protein kinases encoded in the human genome.^1^ These proteins act to regulate many different cellular processes by covalent phosphorylation of serine, threonine or tyrosine amino acid residues of downstream‐effector proteins. Increased kinase activity is often associated with disease (in particular with cancer) such that this class of proteins represents major drug targets.^2^ The protein kinase core has a bi‐lobed structure comprising a smaller N‐terminal lobe and a larger C‐terminal lobe connected by a hinge region. In many cases kinase activity is determined by the phosphorylation status of specific threonine, serine or tyrosine residues located within the activation loop that is situated between these two lobes.^3^ One method used to inhibit kinase activity is prevention of phosphorylation within this loop using tyrosine kinase inhibitors (TKIs). The activation loop is flexible and has been shown to adopt different conformations when bound to different classes of inhibitor. Those inhibitors which bind to the adenosine triphosphate (ATP) site in the hinge region are known as type I inhibitors. Type I inhibitors bind to an active conformation of the kinase, in which the activation loop adopts a conformation amenable to phosphorylation. This is commonly known as a ‘DFG in’ conformation due to the configuration of the conserved aspartic acid‐phenylalanine‐glycine motif present at the N‐terminus of the activation loop. In contrast to type I inhibitors, type II kinase inhibitors cause the activation loop to undergo a conformational change exposing a hydrophobic binding pocket which is located adjacent to the ATP binding site. This type II inhibitor‐induced conformation is known as the ‘DFG out’ conformation and involves reorientation of the activation loop such that the phenylalanine and aspartic acid side chains exchange positions, both rotating by ~180°. Type II inhibitors compete indirectly for ATP binding and are thought to bind only to inactive, unphosphorylated kinases.^4^ For this reason, the discovery of type II inhibitors was serendipitous as high‐throughput screens have generally utilised the active, phosphorylated kinase. Increased understanding of this ‘DFG flip’ is expected to aid drug discovery as type II kinase inhibitors have three advantages over type I inhibitors. Firstly, type II inhibitors have a greater degree of specificity than that of type I inhibitors as they target a kinase conformation where the ATP binding site is not compatible with ATP binding.^5^ Secondly, cellular levels of ADP and ATP are in the mM range such that type I inhibitors suffer from efficacy problems which type II inhibitors may circumvent.^6^ Thirdly, wider intellectual property space is available for commercial exploitation of type II inhibitors.

The first kinase shown to adopt a ‘DFG out’ conformation was ABL kinase upon binding to Imatinib/Gleevec, a small molecule approved for the treatment of chronic myelogenous leukaemia.^7^ Since this discovery, additional kinases have been found by use of X‐ray crystallography to adopt an inactive, ‘DFG out’ conformation upon type II inhibitor binding, e.g. VEGFR,^8^ P38α MAPK,^9^ LCK, MET, KIT, PDGFR, cSRC,^10^ BRAF1, P38 MAPK, and VEGFR2.^11^ A report by Tucker et al^12^ demonstrates using X‐ray crystallography that fibroblast growth factor receptor 1 (FGFR1) is able to adopt a ‘DFG out’ conformation upon binding to the Ponatinib inhibitor. FGFR1 is a membrane‐associated receptor tyrosine kinase which plays crucial roles in human development. Over‐activation or amplification of FGFR1 has been found to occur in many different cancers including oral squamous cell carcinoma,^13^ breast cancer,^14^ and lung cancer.^15^, ^16^

Currently, the gold standard used to determine whether an inhibitor binds to a kinase in the ‘DFG in’ or the ‘DFG out’ conformation has been X‐ray crystallography or nuclear magnetic resonance (NMR) analysis. Both these techniques require significant amounts of protein and are time‐consuming. In addition, X‐ray crystallographic determination of the DFG loop status is not trivial due to the flexibility of the DFG loop region which often has poorly defined or absent electron density.^17^ Rabuck et al have identified that type I and type II kinase inhibitors of ABL kinase can be distinguished by unfolding experiments performed in conjunction with electrospray ionisation ‐ ion mobility spectrometry ‐ mass spectrometry (ESI‐IMS‐MS).^18^ Here, we show that this technique can be applied to identify novel inhibitors of FGFR1 kinase and also to distinguish between type I and type II inhibitors. As the FGFR1 kinase domain can be expressed recombinantly and purified from *E. coli*, and the fact that crystallisation protocols have been established,^19^, ^20^ we sought to develop this ESI‐IMS‐MS method as a rapid test to determine whether FGFR1 has adopted the ‘DFG out’ or ‘DFG in’ kinase conformation in the presence of ligands. Given the structural similarity between VEGFR(2) and FGFR1,^21^ we hypothesised that inhibitors which induce a ‘DFG out’ conformation upon binding to VEGFR(2) might also induce ‘DFG out’ in FGFR1. We present isothermal titration calorimetry (ITC), surface plasmon resonance (SPR) and microscale thermophoresis (MST) data to demonstrate solution‐phase inhibitor affinity for FGFR1. Protein unfolding experiments were performed on the FGFR1–inhibitor complexes using ESI‐IMS‐MS analysis, showing different FGFR1 unfolding patterns depending on the ‘DFG in’ or ‘DFG out’ status. Thus, we have demonstrated the potential for ESI‐IMS‐MS to be used as a fast, screening method to aid the discovery of novel type II inhibitors of FGFR1 kinases, with the associated benefits that ESI‐IMS‐MS requires low μM protein concentrations and has a rapid analysis time.

## 2 MATERIALS AND METHODS

### 2.1 Protein expression and purification

Human FGFR1 consisting of residues 457–765 with C488A and C584S mutations was prepared as described by Norman et al.^20^ The two surface cysteine mutations reduce the level of disulphide‐linked FGFR1 aggregates.^19^

### 2.2 Compounds

Ponatinib used for FGFR1 co‐crystal structure determination was obtained from Sequoia Research Products (Pangbourne, UK). Ponatinib, Sorafenib, and Linifanib for ESI‐IMS‐MS experiments were purchased from Selleckchem (Newmarket, UK). Compounds A to E were synthesised and provided by AstraZeneca (Alderley Park, Cheshire, UK). JK‐P3 and JK‐P5 were synthesised as described and provided by Dr Jayakanth Kankanala (University of Leeds, UK).^22^

### 2.3 X‐ray crystallography

Protein and crystals were obtained according to an established procedure.^19^ Crystals were soaked in JK‐P3 and JK‐P5 (1 mM each) compounds in mother liquor containing 2% dimethyl sulfoxide (DMSO) at 4°C for 14 days and 1 day, respectively. Crystals were flash cooled in a stream of nitrogen gas at 100 K (Oxford Cryostreams Ltd, Oxford, UK) prior to diffraction data collection at 100 K. Data for JK‐P3‐soaked crystals were collected on beamline I02 at the Diamond synchrotron (Didcot, Oxfordshire, UK) using a Q315 CCD detector (ADSC, Poway, CA, USA). JK‐P5 was collected using CuKα radiation from a FRE rotating anode generator (Rigaku, Sevenoaks, UK) equipped with VariMaxHF optics, a Saturn944 CCD detector and an XStream cryo‐cooling system (Rigaku). Data were integrated and scaled using XDS^23^ and SCALA^24^ as implemented within the autoPROC software package.^25^ Data reduction and structure solution by molecular replacement were carried out using programs from the CCP4 software suite.^26^ Compounds JK‐P3 and JK‐P5 were modelled into the electron density using Flynn analysis.^27^ The protein–inhibitor complex model was refined using Buster,^28^ interspersed with rounds of manual model building in Coot.^29^ The final structures have been deposited in the Protein Data Bank with the PDB codes 4uwc and 4uwb for JK‐P3 and JK‐P5, respectively, together with structure factors and detailed experimental conditions. Structures for Ponatinib are described in Tucker et al,^12^ with PDB codes 4v01 and 4v04. Detailed statistics of the data collection and final model are presented in Figure S1 (supporting information). All structural figures were drawn using PyMOL.^30^

### 2.4 Isothermal titration calorimetry (ITC)

ITC was carried out using a Microcal ITC_200_ instrument (GE Healthcare Biosciences, Amersham, UK). FGFR1 was dialysed against the experimental buffer (20 mM Tris/HCl, 100 mM NaCl and 5 mM DTT pH 7.8) prior to titration with inhibitor. Inhibitors were dissolved in DMSO, and experimental buffer DMSO concentration was matched. Titrations were performed at 25°C with 2 μL injections of inhibitor (200 μM) every 180 s into the cell containing FGFR1 (20 μM) with stirring at 1000 rpm. Results were analysed in Origin (OriginLab, Northampton, MA, USA) and fitted using a one‐site binding model. Representative ITC curves are shown in Figure S2 (supporting information).

### 2.5 Surface plasmon resonance (SPR)

SPR experiments were performed using a Biacore S51 biosensor (GE Healthcare Life Sciences, Amersham, UK). Series S NTA (nitrolotriacetic acid) sensor chips (GE Healthcare Life Sciences) were used. All experiments were carried out using phosphate‐buffered saline (PBS) pH 7.4, EDTA (50 μM), 0.05% Surfactant P20 (v/v) and 5% DMSO (v/v) as running buffer. Compound stocks in DMSO were diluted in DMSO to concentrations 20‐fold higher than the final assay concentration. Finally, they were diluted 1:20 (v/v) in PBS pH 7.4, EDTA (50 μM), 0.05% Surfactant P20 (v/v) to achieve the target concentration resulting in a final DMSO concentration of 5% (v/v).

Unphosphorylated, histidine‐tagged FGFR1 was immobilised as the ligand onto NTA sensor chips using a capture coupling method.^30^ PBS pH 7.4, EDTA (50 μM), 0.05% Surfactant P20 (v/v) was used as immobilization buffer. The NTA surface was first activated with a 2 min injection of NiSO_4_ (500 μM) in running buffer before the carboxymethyl dextran surface was activated with a 7 min injection of a 1:1 ratio of 1‐ethyl‐3‐(3‐dimethylaminopropyl) carbodiimide (EDC; 0.4 M) and 0.1 M *N*‐hydroxysuccinimide (NHS). His‐tagged protein was diluted into running buffer to a concentration of 30 μg mL^−1^ and immobilised to the surface with a 7 min injection. Remaining activated groups were blocked with a 7 min injection of Tris (0.1 M), pH 8.0. The resulting immobilization level was 6400 resonance units (RU).

For inhibitor–FGFR1 interactions the binding affinities (*K*_*d*_) were determined from dosage experiments that were carried out at a constant flow rate of 90 μL min^−1^ in running buffer at 25°C. Compounds were tested at nine different concentrations and individual concentrations were injected in triplicates from lowest to highest concentration for 60 s association and 600 s dissociation time. Zero‐buffer blank injections and DMSO calibrations were included for double referencing. Equilibrium analysis was performed by fitting the binding responses at equilibrium to a 1:1 steady‐state affinity model available within Scrubber 2 software. Representative sensorgrams are shown in Figure S3 and *K*_*d*_ plots in Figure S4 (supporting information).

### 2.6 Microscale thermophoresis (MST)

Thermophoresis was used to measure the binding interactions between FGFR1 and compounds. The MST measurements were performed using a Monolith NT.LabelFree instrument (NanoTemper Technologies GmbH, Munich, Germany). In this instrument, an infrared laser beam and light (fluorescence excitation and emission) was coupled with a dichroic mirror and focused on the sample. When the IR‐laser was on, it heated a small area of the sample and created a temperature gradient. The total fluorescence of the focused area was measured when the IR‐laser was off (Fcold) and on (Fhot). The capillaries were filled with the sample (less than 5 μL was required for each capillary). The following MST settings were used: LED power 70%, MST laser power 40%, fluorescence before (5 s), MST on (30 s), fluorescence after (3 s). The temperature of the instrument was set to 25°C for all measurements. After the capillary scan thermophoresis FGFR1 was measured for 30 s in the presence of the varied concentrations of compound. Fnorm = Fhot/Fcold was analysed and plotted by NT Analysis software (NanoTemper Technologies GmbH). Fraction bound = (Fnorm – Fnorm [unbound])/(Fnorm (bound) – Fnorm (unbound)) was calculated and the binding data were fitted to a Hill function (n = 1) using the instrument analysis software. To eliminate artifacts caused by labelling or modifying proteins, the fluorescence of tryptophan residues was used to monitor the local protein concentration. For each compound, a titration series with constant protein concentration and varying compound concentrations was prepared in a final solution of 1% DMSO. Potential autofluorescence of each ligand was checked with no fluorescence signal detected for the compounds. The final protein concentration was 60 nM. Representative ITC curves are shown in Figure S5 (supporting information).

### 2.7 ESI‐IMS‐MS

FGFR1 (2 μM) and inhibitors (8 μM) were incubated separately in ammonium acetate (100 mM) for 1 h at room temperature prior to analysis. A Synapt G2‐S travelling‐wave ion mobility spectrometry ‐ mass spectrometer equipped with a nano‐ESI source was used for all sample analyses (Waters Corp., Manchester, UK). Samples were analysed by nano‐ESI from in‐house fabricated gold–palladium‐coated borosilicate capillaries assembled using a P‐97 micropipette puller (Sutter Instrument Co., Novato, CA, USA) and a sputter coater (Polaron SC7620; Quorum Technologies Ltd, Kent, UK). The following instrument parameters were set: capillary voltage 1.8 kV, cone voltage 30 V, source temperature 100°C, backing pressure 9.3 mbar, travelling‐wave height 40 V, travelling‐wave speed 500 m/s, IMS He gas flow rate 150 mL/min, IMS N_2_ gas flow rate 50 mL/min. For the collision‐induced unfolding (CIU) experiments, the trap voltage was increased to 21 V or 24 V and the transfer voltage was maintained at 4 V. Data were processed by use of the MassLynx (version 4.1) and Driftscope software supplied with the mass spectrometer. The *m/z* scale was calibrated using a separate introduction of aqueous CsI at 1 mg/mL.

## 3 RESULTS

### 3.1 X‐ray crystallography confirms FGFR1 inhibitor binding modes as type I or type II based upon the DFG loop status

**FIGURE 1:**
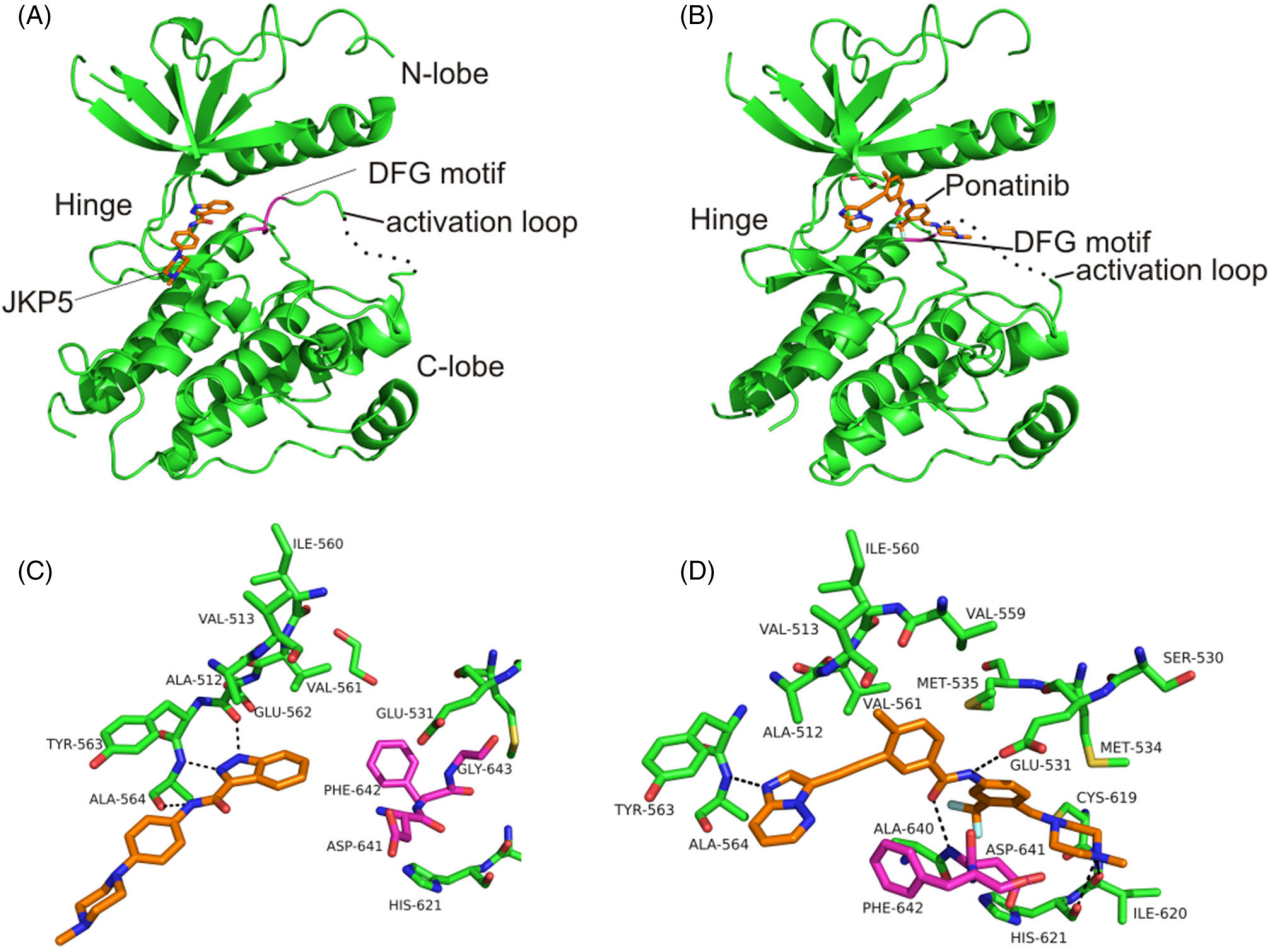
X‐ray crystal structures of FGFR1 kinase domain in complex with a, c, JKP5 (PDB 4uwb), a type I TKI, and b, d, Ponatinib (PDB 4v01), a type II TKI. The activation loop can be seen where the location of the DFG motif is highlighted in magenta. JK‐P5 and Ponatinib are drawn with carbon atoms in orange whilst FGFR1 carbon atoms are green or pink in the case of the DFG motif. Hydrogen‐bond interactions are shown as dotted lines

**FIGURE 2:**
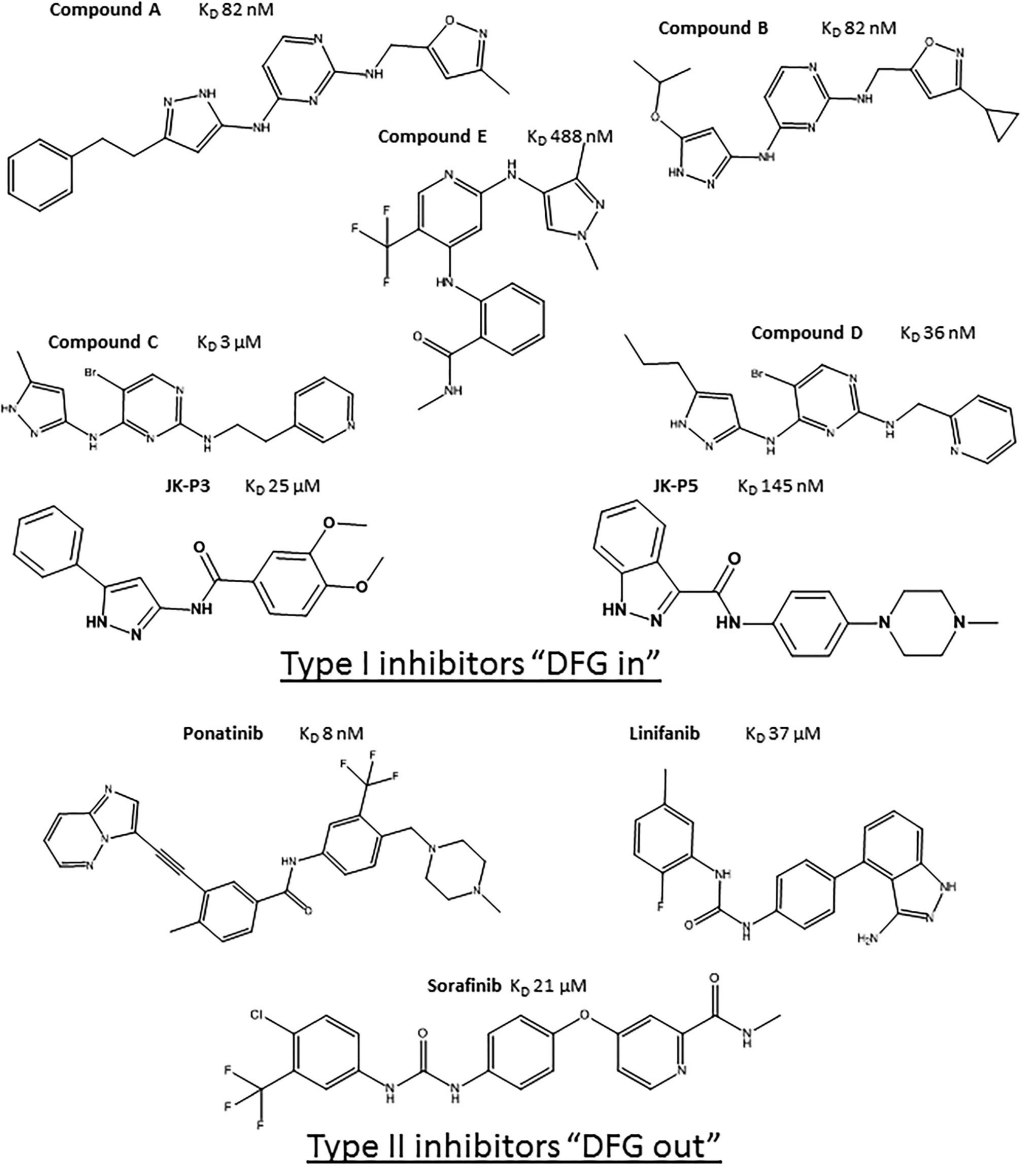
FGFR1 inhibitors and their binding affinities for FGFR1. *K*_*d*_ values for type I TKIs were measured by ITC and type II *K*_*d*_ values were measured by SPR. (Note: Sorafenib showed inconclusive crystal density for the orientation of DFG loop to be determined)

First, to define unambiguously whether the compounds used in this developmental study were type I or type II inhibitors of FGFR1, the X‐ray crystal structures of the FGFR1–ligand complexes were determined. Figure 1 shows examples of type I and type II inhibitors binding to the protein FGFR1. The crystallography data confirmed the binding modes for all, bar Sorafinib, of the FGFR1 inhibitors used in this study as well as the orientation of the DFG motif. Of the ten compounds tested (Figure 2), seven bind to the ‘DFG in’ kinase conformation (compounds A to E, JK‐P3, and JK‐P5) while two induce a ‘DFG out’ conformation (Ponatinib and Linifanib) (see Norman et al^20^ for relevant PDB numbers). It was not possible to determine the DFG status of the Sorafenib co‐crystal structure due to the poor crystal density surrounding the DFG loop. JK‐P3 and JK‐P5 were originally designed to bind to VEGFR kinase using the in silico de novo design programme SPROUT.^22^ Both JK‐P3 and JK‐P5 were also found to have cellular activity against FGFR1.^22^ The FGFR1 co‐crystal structures presented here are in good agreement with the predicted binding poses from SPROUT, with three hydrogen bonds observed between the JK‐P5 inhibitor and residues Glu562 and Ala564 of the FGFR1 hinge region (Figures 1A and 1C). Specifically, the indazole (JK‐P5) or pyrazole (JK‐P3) nitrogen atoms are involved in hydrogen bonds with the backbone carbonyl of Glu562, and the backbone amide group of Ala564, whilst the amide NH forms a hydrogen bond with the backbone carbonyl of Ala564. JK‐P3 and JK‐P5 were designed to bind in the kinase hinge region of a ‘DFG in’ conformation.

Sorafenib, a known type II inhibitor of VEGFR kinase,^8^ has been shown to inhibit FGFR1 with an *in vitro* IC_50_ of 580 nM.^31^ Given the high degree of structural homology between VEGFR and FGFR1, we hypothesised that Sorafenib could also act as a type II inhibitor of FGFR1. Other type II VEGFR inhibitors include Linifanib,^32^ which similarly induces a DFG out conformation in FGFR1. Figures 1B and 1D show the binding of Ponatinib to FGFR1, highlighting the canonical ‘DFG out’ binding mode in which the bridging amide is engaged in hydrogen bonding with the side‐chain carboxyl group of Glu531 from the kinase α‐C helix and the backbone amide of Asp641 from the DFG motif (adapted from Tucker et al^12^). Here FGFR1 is shown to be able to adopt a ‘DFG out’ conformation upon binding to Linifanib and Ponatinib, making both these ligands type II tyrosine kinase inhibitors (TKIs).

### 3.2 Type I and type II inhibitors show a range of affinities for FGFR1

The range of affinities spanned by the inhibitors selected for this study was determined using ITC, SPR and MST, to determine if binding affinity was related to binding mode. Association constants for the type I TKIs were measured using ITC or MST (Figure 2 presents the *K*_*d*_ values; Figure S2 shows the ITC binding isotherms and Figure S5 the MST data, supporting information). Type II inhibitors were not, in our hands, amenable to analysis by ITC; therefore, SPR was used to obtain dissociation constant (*K*_*d*_) information for these ligands (see Figures S3 and S4, supporting information). The affinities of both type I and type II TKIs for FGFR1 were found to span the nM to μM range. The type I TKI with the highest affinity for FGFR1 was compound D with a *K*_*d*_ of 36 nM whilst the highest affinity type II TKI was Ponatinib with a *K*_*d*_ of 8 nM. The type I inhibitor with the lowest affinity was JK‐P3 with a *K*_*d*_ value for FGFR1 of 25 μM; the weakest type II inhibitor was Linifanib with a *K*_*d*_ value of 37 μM. The similarity in range of affinities measured for the type I and type II TKIs used in this study provides confidence that the effects of affinity from those of conformational change can be decoupled when interpreting the ESI‐IMS‐MS unfolding data/profiles.

### 3.3 ESI‐MS analysis of type I and type II inhibitors binding to FGFR1

**FIGURE 3:**
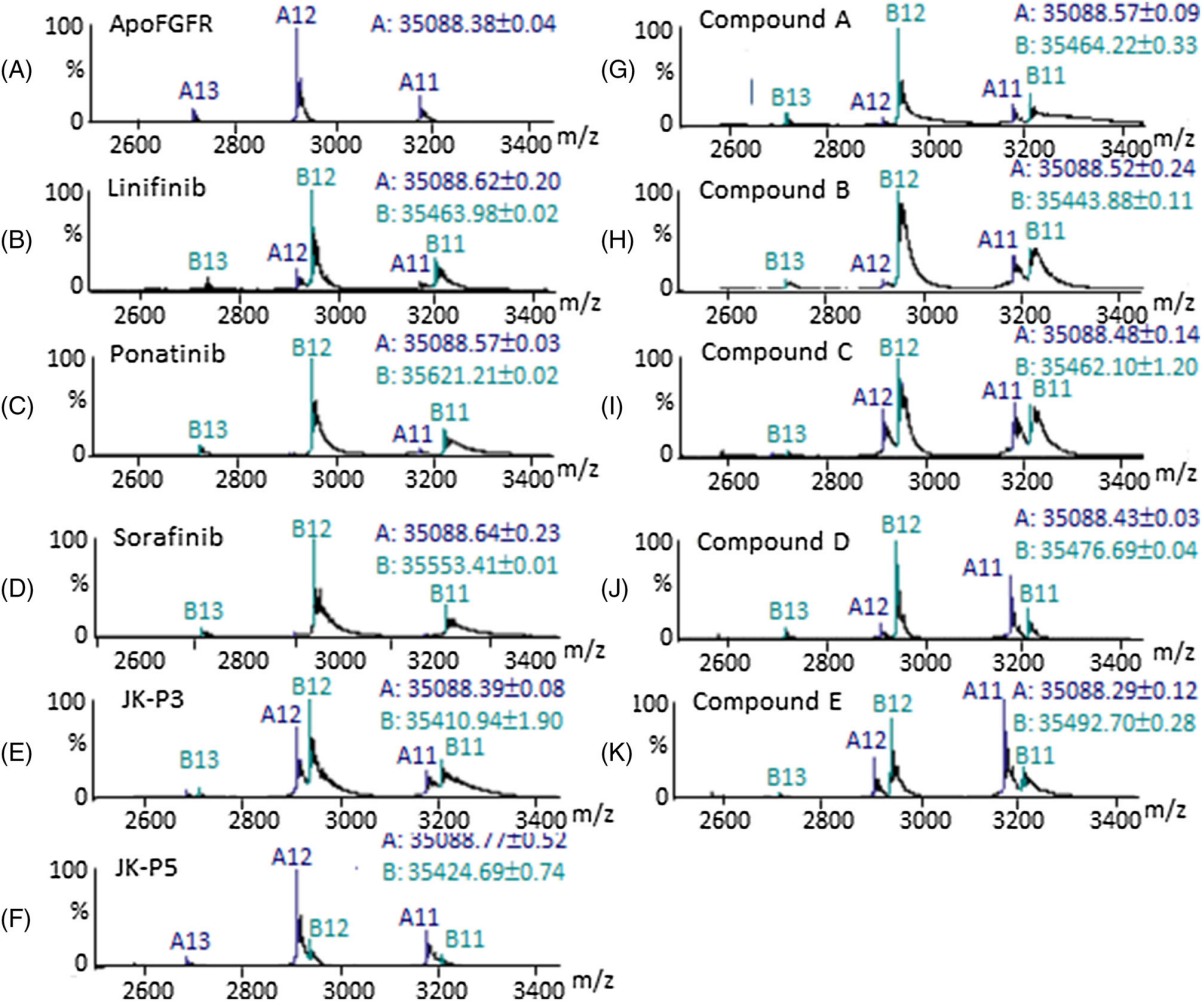
ESI‐MS spectra of FGFR1 alone and in the presence of an inhibitor: a, *apo‐*FGFR1; b–k, FGFR1 bound to Linifinib, Ponatinib, Sorafinib, JK‐P3, JK‐P5, compound A, compound B, compound C, compound D and compound E, respectively. In each spectrum 'A' refers to the unbound protein and 'B' to the protein bound to one molecule of ligand. The numbers following 'A' and 'B' refer to the number of positive charges carried on the ions. The molecular masses of A and B are shown in the top right of each spectrum; for full mass measurement details, see Figure S6 (supporting information)

ESI‐MS has proven to be a valuable tool in structural biology allowing the preservation of non‐covalently bound protein–ligand complexes in the gas phase and providing the ability to determine information about the conformation of a protein from its charge‐state distribution.^33^ ESI‐MS analysis of FGFR1 from a neutral solution of ammonium acetate results in a narrow charge state range of 11+, 12+, and 13+ ions in a single Gaussian distribution (Figure 3A). Such a narrow charge‐state distribution indicates that the protein has maintained its folded conformation throughout the ESI‐MS analysis; a partially folded or unfolded protein would be expected to have a much wider range of charges, including more highly charged ions which often accommodate more than one Gaussian distribution. The mass measured from these charge states is consistent with the calculated mass for this protein (Figure S6, supporting information).

Non‐covalent binding of a single molecule of both type I and type II TKIs to FGFR1 was detected for all ten ligands in this study using ESI‐MS (Figures 3B–3K), with the charge‐state distribution remaining compact, thus indicating the protein is still occupying a folded conformation. The mass measurements for *apo‐*FGFR1 and its ligand‐bound complexes are shown in Figure S6 (supporting information).

### 3.4 Using ESI‐IMS‐MS to monitor protein unfolding

The commercialisation of IMS coupled to MS has taken protein conformational analysis to a higher level.^34^ IMS‐MS allows the separation of ions of the same mass and *m/z*, based on their rotationally averaged collision cross‐section (CCS) or shape^35^; for example, multiple conformations of a protein can be separated and mass measured in a single experiment.

A Synapt G2‐S mass spectrometer was used for these studies. Briefly, following ESI of the analyte, the sample ions pass through an ion guide into the first, quadruple, analyser, and then enter the Triwave unit. The Triwave consists of a trap cell followed by a travelling‐wave ion mobility separation device and finally a transfer cell. On exiting the latter, the sample ions are transmitted through the final, time‐of‐flight, analyser before detection. Protein unfolding can also be monitored with this instrument by use of collision‐induced unfolding (CIU). Here, the protein ions are subjected to high‐energy collisions with the argon gas contained in the trap collision cell, immediately prior to the IMS cell. The resulting increase in CCS brought about by the protein unfolding can then be monitored using IMS‐MS. If multiple protein conformations are present, these will be separated at the IMS stage as the more extended protein ions will have larger CCSs than the more compact, folded protein ions and thus will take longer to traverse the IMS cell.^36^

**FIGURE 4:**
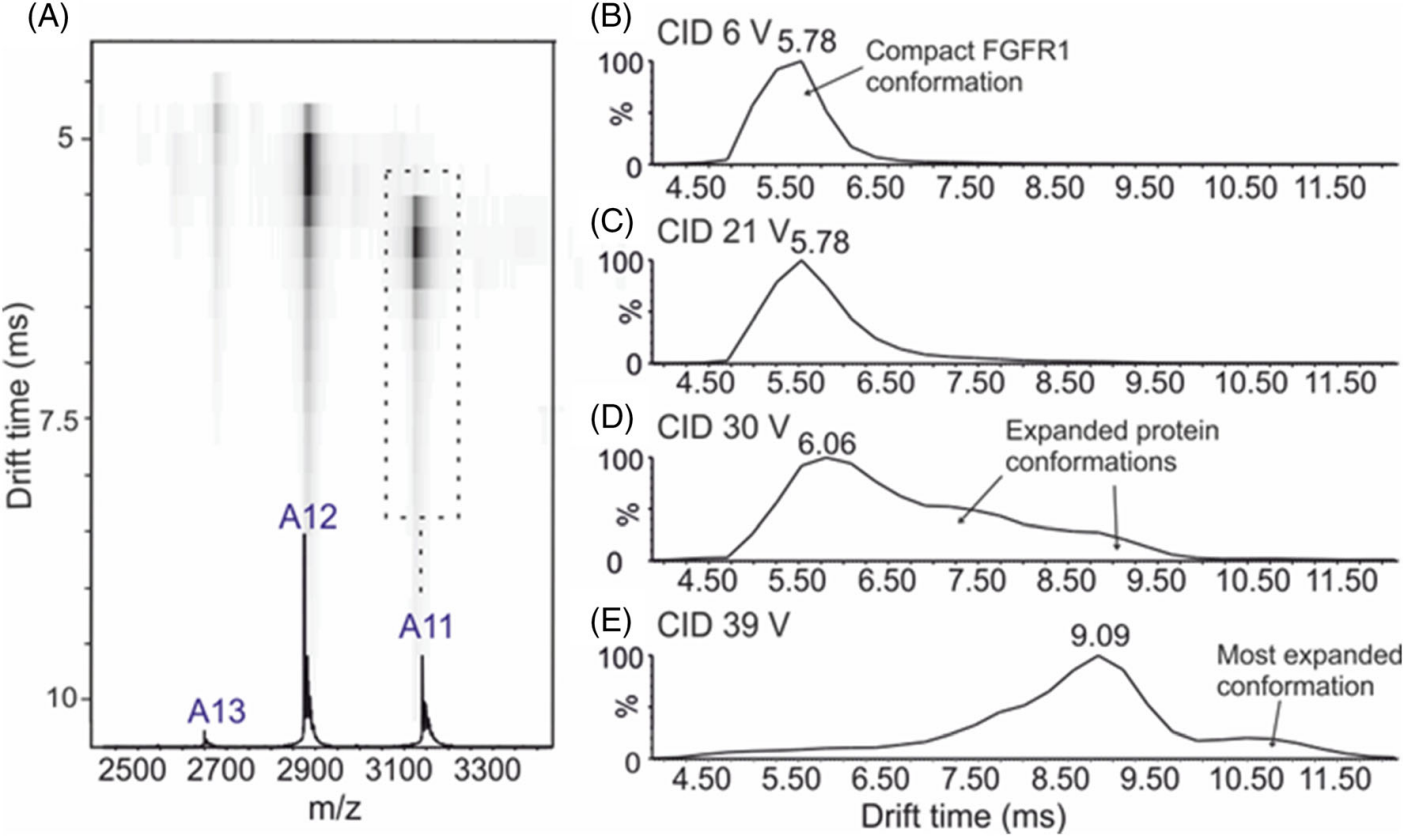
ESI‐IMS‐MS data: a, Driftscope plot of *apo*‐FGFR1 at 6 V trap voltage. The *m/z* value is shown on the x‐axis and the IMS drift times of the ions on the y axis. The drift times of the 11+ *apo*‐FGFR1 ions at b, 6 V; c, 21 V; d, 30 V; and e, 39 V trap energies show a shift to extended profiles at longer drift times indicating CIU of the protein when subjected to higher instrumental voltages

The molecular mass and the CCS of a protein, or protein complex, can both be measured in a single experiment. Figure 4 shows the ESI‐IMS‐MS analysis of *apo*‐FGFR1. The Driftscope plot (Figure 4A) presents the MS and IMS data simultaneously, with the *m/z* values on the x‐axis and the IMS drift times on the y axis; the shaded areas represent the ion intensities. Figure 4B presents these data as a profile of the drift time. Increasing the trap energy prior to the IMS cell results in CIU (Figures 4C–4E), illustrating how the protein unfolding process can be monitored using IMS‐MS, with the more extended, less folded, protein ions having longer drift times owing to their larger CCS (Figure 4E).

### 3.5 ESI‐CIU‐IMS‐MS unfolding experiments suggest different unfolding pathways for FGFR1 when bound to type I or type II inhibitors

To see if type I and type II inhibitors affect the conformational profile of FGFR1, the protein was incubated with each inhibitor separately prior to nano‐ESI‐CIU‐IMS‐MS analysis. All charge‐state ions arising from the FGFR1–ligand complexes (selected individually by the initial quadrupole analyser) were subjected to CIU‐induced unfolding in the trap prior to IMS separation. During CIU, the protein–ligand *m/z* peak intensity was found to be reduced and the amount of unbound protein was seen to increase, especially at the higher 21 V and 24 V trap voltages used, indicating that some bound ligand was being stripped from the protein under these conditions.

**FIGURE 5:**
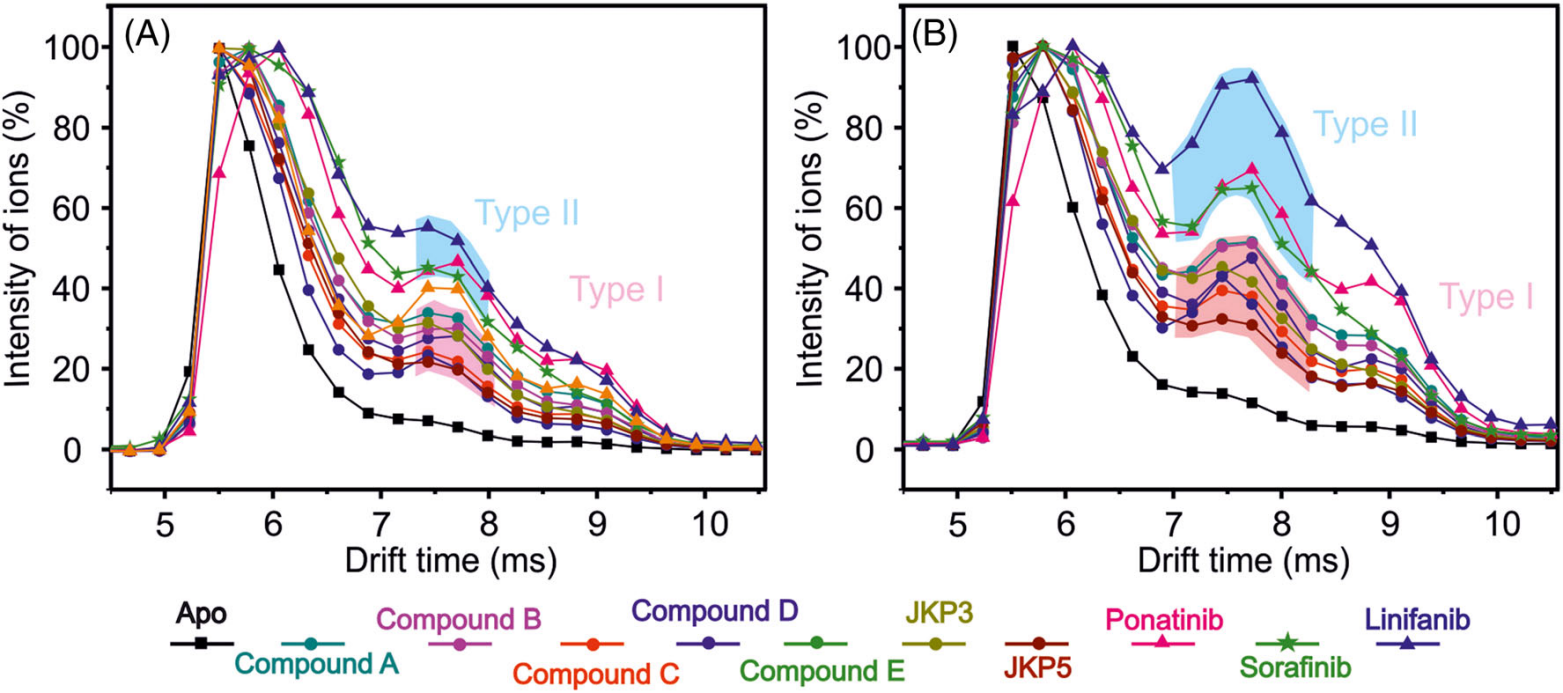
Drift time profiles of the 11+ FGFR1 charge‐state ions subjected to a, 21 V trap and b, 24 V trap current after incubation with type I inhibitors (circles), type II inhibitors (triangles), or no inhibitor (black squares). Each point is the average of three replicate datasets. Differences between the FGFR1 unfolding profiles are highlighted in pink (indicative of type I inhibitor binding) and blue (type II inhibitor binding)

On inspection of the IMS data of the unbound 11+ ions (chosen as the lowest charge state of FGFR1 and therefore deemed to be most representative of the native protein state), which consist of *apo*‐protein and ligand‐stripped protein, a different profile for the type I and the type II inhibitors is clearly observed (Figure 5).^37^ CIU gas‐phase experiments indicate that the 11+ FGFR1 ions behave in a different manner depending on whether bound to type I or type II inhibitors. During these analyses, the trap energy was increased and protein unfolding was monitored by examining the drift times of the protein ions during their transit through the IMS cell. At both 21 V and 24 V trap energies there are two dominant populations of FGFR1 conformations, a more compact conformation with a drift time of 5.5 ms consistent with the *apo* protein and a more extended conformation at 7.6 ms.

It can be seen from the data in Figure 5 that type II inhibitors consistently cause FGFR1 to populate the more unfolded conformation to a greater extent than do the type I inhibitors. At 21 V, in the presence of type I inhibitors, the 7.6 ms FGFR1 conformer has an intensity of between 22–35% of the total ion current, whereas, in the presence of type II inhibitors, FGFR1 populates this conformer at an intensity of greater than 40%. At 24 V, in the presence of type II inhibitors, FGFR1 populates the 7.6 ms conformer at between 64–95% whereas the type I inhibitors populate this conformer at 30–50%. The average intensities of the components appearing at 7.6 ms drift time, compared with the base peak at 5.5 ms representing the most compact protein conformation, are 27.6% (s.d. 3.9) for type I inhibitors and 48.9% (s.d. 4.4) for type II inhibitors at 21 V trap current. At 24 V trap current, the average intensities are 44.2% (s.d. 5.6) and 76.7% (s.d. 11.6) for type I and type II inhibitors, respectively (Figure S7, supporting information). For both trap currents, the measurements for the type I and type II inhibitors are clearly separated and the classification of each inhibitor is unambiguous.

Thus, the FGFR1 CIU unfolding profile can be used to detect whether FGFR1 is bound to a type I or type II inhibitor.

It was not possible to determine the binding mode of the Sorafenib–FGFR1 complex by X‐ray crystallography, which is a common feature of type II TKIs due to the dynamic nature of the DFG loop. The ESI‐IMS‐MS experiments presented show Sorafenib to have a CIU profile akin to that of the type II inhibitors and this ligand can now be classified as such with confidence. Hence, ESI‐IMS‐MS has proved to be a valuable screening method for defining type I and type II inhibitors of FGFR1.

## 4 DISCUSSION

The data presented show that FGFR1 can adopt an inhibitor‐induced ‘DFG out’ conformation and that ESI‐IMS‐MS can be used successfully to distinguish between the ‘DFG in’ and ‘DFG out’ ligand‐bound conformations of FGFR1. Using 21 and 24 V trap voltages to induce protein unfolding resulted in the generation of two protein conformers, with IMS drift times of 5.5 and 7.6 ms, which were clearly separable in the IMS cell.

The 7.6 ms conformer is only very lowly populated in the case of *apo‐*FGFR1. It is likely that the population of this more unfolded conformer is increased by the dissociation of both type I and type II inhibitors from the protein. Further, type II inhibitors exhibit a greater increase in the population of the 7.6 ms conformer compared with type I inhibitors. As both the type I and type II TKIs used in this study have been shown to span a range of affinities for FGFR1 it is unlikely that the increase in this conformer is due simply to the classes having differing binding propensities.

A possible explanation for the increased levels of the 7.6 ms conformer in the presence of type II inhibitors is that FGFR1 has a lower stability when in the ‘DFG out’ conformation and therefore is capable of unfolding to a greater extent compared with the ‘DFG in’ conformation. Given that the crystal structure of *apo*‐FGFR1 shows the activation loop to adopt the ‘DFG in’ conformation, this is likely to be the most energetically favourable conformation.^19^ To date, ~70% of known kinase structures adopt the ‘DFG in’ conformation, while ~22% are in an intermediate state and ~3% adopt a ‘DFG‐out’ conformation in the absence of ligand.^38^ The dynamics of the ‘DFG flip’ are not fully understood; however, it is thought that certain kinases are able to convert readily between ‘DFG in’ and ‘DFG out’ conformations.^39^ Not all kinases adopt a ‘DFG out’ conformation in the absence of ligand and this is thought to be due to the free energy penalty incurred by the protein on adopting the ‘DFG out’ conformation. Indeed, Gleevec targets ABL kinase preferentially over cSrc kinase due to the higher free energy penalty incurred by cSrc in adopting the ‘DFG out’ conformation.^17^, ^40^

Traditionally, X‐ray crystallography and NMR spectroscopy have been the only techniques available which could determine whether an inhibitor binds to the ‘DFG in’ or ‘DFG out’ kinase conformation. Both these techniques are time‐consuming and require significant amounts of protein. Conversely, ESI‐IMS‐MS requires low μM concentrations of protein and, in combination with an automated nano‐ESI infusion device, could allow IMS‐MS to be used as a high‐throughput screening method.

Rabuck et al identified that IMS‐MS unfolding can be used to identify whether ABL‐kinase was bound to a type I or a type II TKI.^18^ Here we have shown, with supporting biochemical data, that this technique can be applied to FGFR1 kinase to identify a range of recognised and novel kinase inhibitors.

## Supporting information


**Figure S1. Refinement statistics for X‐ray crystallography structures.** For Ponatinib details see: Tucker J, Klein T, Breed J, Breeze AL, Overman R, Phillips C, Norman RA. Structural insights into FGFR kinase isoform selectivity: diverse binding modes of AZD4547 and ponatinib in complex with FGFR1 and FGFR4. *Structure.* 2014:**22**(12):1764–1774.
Figure S2. ITC curves for FGFR1 inhibitors.
Compound A; (b) Compound B; (c) Compound C; (d) Compound D; (e) Compound E.
**Figure S3. Surface Plasmon Resonance Data.** Sensorgrams for inhibitors interacting with a 6,400‐RU FGFR1 surface. The highest concentration for each compound was as follows: (a) Sorafenib, 160 μM; (b) Linifanib, 500 μM. Each compound was injected over a 256‐fold concentration range using a 2‐fold dilution series. Each concentration was injected three times. The compound structure, name, and molecular mass are provided on each data set.
**Figure S4. Plots of binding data**. Data normalized to percent capacity as a function of inhibitor concentration for inhibitor‐FGFR1 interactions. (a) Fit of the triplicate Sorafenib equilibrium response data from the His‐FGFR1 surface to a 1:1 interaction; (b) Fit of the triplicate Linifanib equilibrium response data from the His‐FGFR1 surface to a 1:1 interaction. The compound structure, name, and binding constants (*K*_*D*_ ± SE) are provided on each data set.
**Figure S5. Microscale thermophoresis data.** (a) JK‐P3 and FGFR1 kinase thermophoresis trace; (b) JK‐P5 and FGFR1 kinase thermophoresis trace.
**Figure S6. Mass measurements.** (a) Sequence and mass measurements for *apo*‐FGFR1; (b) table showing mass measurements of *holo*‐FGFR1 when FGFR1 is bound to each inhibitor, together with the derived mass of each ligand (compared with each ligand's calculated mass).
Figure S7. Table showing the intensities of the unfolded protein conformer appearing at drift time ca 7.6 ms for each of the type I and type II ligands. The means and standard deviations have been calculated for both types of inhibitor at 21 V and 24 V trap energies.

## Data Availability

The data that support the findings of this study are available on request from the corresponding author. The data are not publicly available due to privacy or ethical restrictions.
