# Supplementary material for: Validation of ion mobility spectrometry ‐ mass spectrometry as a screening tool to identify type II kinase inhibitors of FGFR1 kinase
Source: Rapid Commun Mass Spectrom. 2021 Jun 29;39(Suppl 1):e9130. doi: 10.1002/rcm.9130 (PMC12062780; doi:10.1002/rcm.9130)
Supplement: Supplementary file 1 — Figure S1. Refinement statistics for X‐ray crystallography structures. For Ponatinib details see: Tucker J, Klein T, Breed J, Breeze AL, Overman R, Phillips C, Norman RA. Structural insights into FGFR kinase isoform selectivity: diverse binding modes of AZD4547 and ponatinib in complex with FGFR1 and FGFR4. Structure. 2014:22(12):1764–1774. Figure S2. ITC curves for FGFR1 inhibitors. Compound A; (b) Compound B; (c) Compound C; (d) Compound D; (e) Compound E. Figure S3. Surface Plasmon Resonance Data. Sensorgrams for inhibitors interacting with a 6,400‐RU FGFR1 surface. The highest concentration for each compound was as follows: (a) Sorafenib, 160 μM; (b) Linifanib, 500 μM. Each compound was injected over a 256‐fold concentration range using a 2‐fold dilution series. Each concentration was injected three times. The compound structure, name, and molecular mass are provided on each data set. Figure S4. Plots of binding data. Data normalized to percent capacity as a function of inhibitor concentration for inhibitor‐FGFR1 interactions. (a) Fit of the triplicate Sorafenib equilibrium response data from the His‐FGFR1 surface to a 1:1 interaction; (b) Fit of the triplicate Linifanib equilibrium response data from the His‐FGFR1 surface to a 1:1 interaction. The compound structure, name, and binding constants (K D ± SE) are provided on each data set. Figure S5. Microscale thermophoresis data. (a) JK‐P3 and FGFR1 kinase thermophoresis trace; (b) JK‐P5 and FGFR1 kinase thermophoresis trace. Figure S6. Mass measurements. (a) Sequence and mass measurements for apo‐FGFR1; (b) table showing mass measurements of holo‐FGFR1 when FGFR1 is bound to each inhibitor, together with the derived mass of each ligand (compared with each ligand's calculated mass). Figure S7. Table showing the intensities of the unfolded protein conformer appearing at drift time ca 7.6 ms for each of the type I and type II ligands. The means and standard deviations have been calculated for both types of inhibito [file RCM-39-e9130-s001.docx]

**Validation of IMS-MS as a screening tool to identify type II kinase inhibitors of FGFR1 kinase**

Helen S. Beeston^1^, Tobias Klein^2^, Richard A. Norman^2^, Julie A. Tucker^2^, Malcolm Anderson^2^, Alison E. Ashcroft^1*^, Geoffrey A. Holdgate^2*^.

Addresses:-

1. Astbury Centre for Structural Molecular Biology & Faculty of Biological Sciences, University of Leeds, Leeds, LS2 9JT, UK.
2. Discovery Sciences, BioPharmaceuticals R&D, AstraZeneca, Alderley Park, Macclesfield, SK10 4TG, UK.

* Corresponding authors.

**Supporting Information**

**Refinement statistics for X-ray crystallography structures**

|  | JK-P5 | JK-P3 |
| --- | --- | --- |
| Space group: | C2 | C2 |
| Cell constants a; b; c (Å) | 208.6 57.3 65.6 90.0 107.6 90.0 | 207.5 58.4 65.9 90.0 107.4 90.0 |
| Resolution limit (Å) | **2.31** | **1.96** |
| Resolution range (Å) | 99.44 2.30 | 29.21 1.96 |
| Completeness overall (%) | 97.26 | 87.8 |
| Reflections, unique | 29915 | 47605 |
| Multiplicity | 3.72 | 3.4 |
| *R*merge _overall_ **^1^** | 0.060 | 0.034 |
| *R*value _overall_ (%) **^2^** | 0.23 | 0.179 |
| *R*value _free_ (%) | 0.27 | 0.206 |
| Non hydrogen protein atoms | 4658 | 4640 |
| Non hydrogen ligand atoms | 50 | 48 |
| Solvent molecules | 193 | 339 |
| R.m.s. deviations from ideal values | |  |
| Bond lengths (Å) | 0.011 | 0.010 |
| Bond angles (º) | 1.5 | 1.03 |
| Average *B* values (Å^2^) | |  |
| Protein main chain atoms | 37 | 49 |
| Protein all atoms | 37 | 51 |
| Ligand | 53 | 57 |
| Solvent | 53 | 57 |
| Φ, Ψ angle distribution for residues **^3^** | |  |
| In most favoured regions (%) | 96.1 | 97.4 |
| In allowed regions (%) | 99.5 | 99.7 |
| Outliers (%) | 0.5 | 0.3 |
| **1** *R*_merge_ = ∑*_hkl_* [( Σ*_i_* \|*I_i_* - ‹*I*›\| )/ Σ*_i_ I_i_*]  **2** *R*_value_ = ∑*_hkl_* \|\|*F*_obs_\| - \|*F*_calc_\|\| / ∑ *_hkl_* \|*F*_obs_\|  *R*_free_ is the cross-validation *R* factor computed for the test set of 5 % of unique reflections  **3** Ramachandran statistics as defined by Molprobity (lovell at al, 2003. Prot struct func & gene 50, 437-450) |  |  |

**Figure S1.** **Refinement statistics for X-ray crystallography structures.** For Ponatinib details see: Tucker J, Klein T, Breed J, Breeze AL, Overman R, Phillips C, Norman RA. Structural insights into FGFR kinase isoform selectivity: diverse binding modes of AZD4547 and ponatinib in complex with FGFR1 and FGFR4. *Structure.* 2014:**22**(12):1764-1774.

**ITC curves for FGFR1 inhibitors**


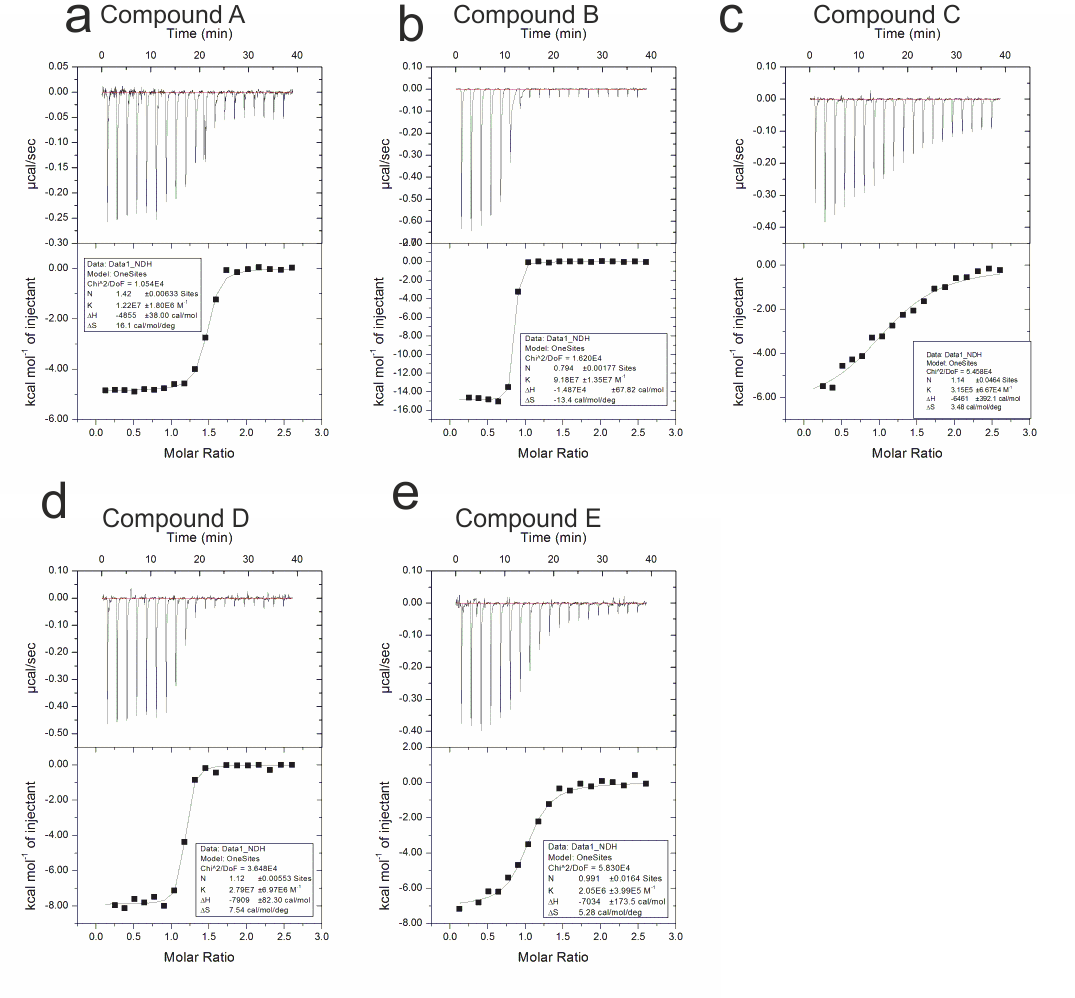


**Figure S2. ITC curves for FGFR1 inhibitors.**

1. Compound A; (b) Compound B; (c) Compound C; (d) Compound D; (e) Compound E.

**Surface Plasmon Resonance Data**

**Figure S3. Surface Plasmon Resonance Data.** Sensorgrams for inhibitors interacting with a 6400-RU FGFR1 surface. The highest concentration for each compound was as follows: (a) Sorafenib, 160 µM; (b) Linifanib, 500 µM. Each compound was injected over a 256-fold concentration range using a 2-fold dilution series. Each concentration was injected three times. The compound structure, name, and molecular mass are provided on each data set.

**Plots of binding data**


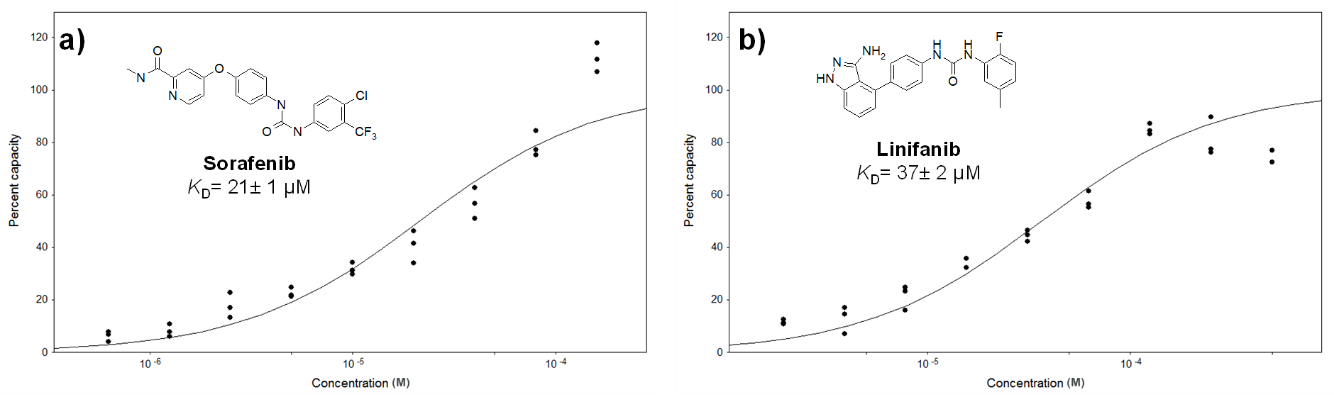


**Figure S4. Plots of binding data**. Data normalized to percent capacity as a function of inhibitor concentration for inhibitor-FGFR1 interactions. (a) Fit of the triplicate Sorafenib equilibrium response data from the His-FGFR1 surface to a 1:1 interaction; (b) Fit of the triplicate Linifanib equilibrium response data from the His-FGFR1 surface to a 1:1 interaction. The compound structure, name, and binding constants (*K_D_* ± SE) are provided on each data set.

**Microscale thermophoresis data**

**(a)**


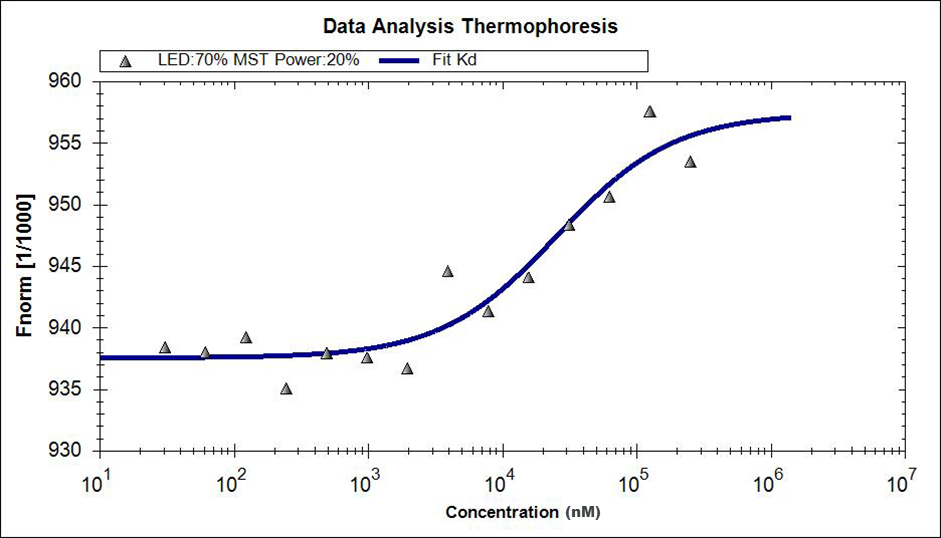


**(b)**


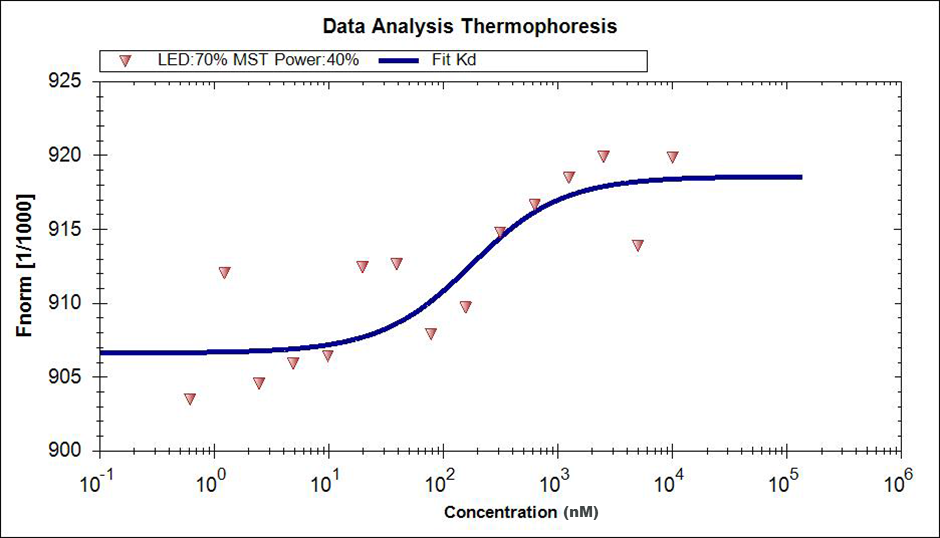


**Figure S5. Microscale thermophoresis data.** (a) JK-P3 and FGFR1 kinase thermophoresis trace; (b) JK-P5 and FGFR1 kinase thermophoresis trace.

**Mass measurements**

1. **Human Fibroblast growth factor receptor 1 (FGFR1) sequence:**

GAGVSEYELP EDPRWELPRD RLVLGKPLGE G**A**FGQVVLAE AIGLDKDKPN RVTKVAVKML KSDATEKDLS DLISEMEMMK MIGKHKNIIN LLGACTQDGP LYVIVEYASK GNLREYLQAR RPPGLEY**S**YN PSHNPEEQLS SKDLVSCAYQ VARGMEYLAS KKCIHRDLAA RNVLVTEDNV MKIADFGLAR DIHHIDYYKK TTNGRLPVKW MAPEALFDRI YTHQSDVWSF GVLLWEIFTL GGSPYPGVPV EELFKLLKEG HRMDKPSNCT NELYMMMRDC WHAVPSQRPT FKQLVEDLDR IVALTSNQE

n.b. Residues Cys488 and Cys584 have been replaced by Ala and Ser residues, respectively, as highlighted in red on the sequence above.

Measured mass: 35,088.45 Da (average of 11 measurements); theoretical mass: 35,088.33 Da.

1. **Table of mass measurements**

| Name | (i). Measured mass (ave.)  i.e. column (iv) – column (iii) (Da) | (ii). Theoretical mass (ave.)    (Da) | (iii). *apo*-FGFR1 measured mass (ave.)  (Da) | (iv). *holo*-FGFR1 measured mass (ave.)  (Da) |
| --- | --- | --- | --- | --- |
| *apo*-FGFR1 |  | 35,088.33 | 35,088.38 |  |
|  |  |  |  |  |
| Linifanib | 375.36 | 375.40 | 35,088.62 | 35,463.98 |
| Ponatinib | 532.64 | 532.56 | 35,088.57 | 35,562.21 |
| Sorafenib | 464.77 | 464.83 | 35,088.64 | 35,553.41 |
| JK-P3 | 322.55 | 323.35 | 35,088.39 | 35,410.94 |
| JK-P5 | 335.92 | 335.40 | 35,088.77 | 35,424.69 |
| Compound A | 375.65 | 357.43 | 35,088.57 | 35,464.22 |
| Compound B | 355.36 | 355.40 | 35,088.52 | 35,443.88 |
| Compound C | 373.62 | 374.24 | 35,088.48 | 35,462.10 |
| Compound D | 388.21 | 388.27 | 35,088.48 | 35,476.69 |
| Compound E | 404.41 | 404.39 | 35,088.29 | 35,492.70 |

**Figure S6. Mass measurements.** (a) Sequence and mass measurements for *apo*-FGFR1; (b) table showing mass measurements of *holo*-FGFR1 when FGFR1 is bound to each inhibitor, together with the derived mass of each ligand (compared with each ligand’s calculated mass).

**IMS-MS Collision Induced Unfolding data analysis.**

| **Protein + ligand** | **IMS-MS Drift**  **Time (ms)** | **Intensity vs**  **base peak (5.5 ms) (%)** |
| --- | --- | --- |
| **21 V Trap** |  |  |
| **Apo FGFR1** | 7.5 | 7.5 |
| **Type I** |  |  |
| Compound A | 7.5 | 34.2 |
| Compound B | 7.5 | 29.2 |
| Compound C | 7.5 | 25.0 |
| Compound D | 7.5 | 27.5 |
| Compound E | 7.5 | 24.2 |
| JKP3 | 7.5 | 30.8 |
| JKP5 | 7.5 | 22.1 |
|  |  | **Mean = 27.6 %**  **s.d. = 3.9** |
| **Type II** |  |  |
| Ponatinib | 7.9 | 46.7 |
| Linifanib | 7.5 | 55.0 |
| Sorafenib | 7.5 | 45.0 |
|  |  | **Mean = 48.9 %**  **s.d. = 4.4** |
|  |  |  |
| **24 V Trap** |  |  |
| **Apo FGFR1** | 7.5 | 13.6 |
| **Type I** |  |  |
| Compound A | 7.7 | 50.8 |
| Compound B | 7.7 | 50.8 |
| Compound C | 7.5 | 40.7 |
| Compound D | 7.7 | 43.2 |
| Compound E | 7.5 | 45.8 |
| JKP3 | 7.5 | 45.8 |
| JKP5 | 7.5 | 32.2 |
|  |  | **Mean = 44.2 %**  **s.d. = 5.6** |
| **Type II** |  |  |
| Ponatinib | 7.7 | 71.2 |
| Linifanib | 7.7 | 92.8 |
| Sorafenib | 7.5 | 66.1 |
|  |  | **Mean = 76.7 %**  **s.d. = 11.6** |

Figure S7. Table showing the intensities of the unfolded protein conformer appearing at drift time ca 7.6 ms for each of the type I and type II ligands. The means and standard deviations have been calculated for both types of inhibitor at 21 V and 24 V trap energies.
